# A review of attention-deficit/hyperactivity disorder from the perspective of brain networks

**DOI:** 10.3389/fnhum.2013.00192

**Published:** 2013-05-15

**Authors:** Angelica De La Fuente, Shugao Xia, Craig Branch, Xiaobo Li

**Affiliations:** ^1^Ferkauf Graduate School of Psychology, Albert Einstein College of Medicine, Yeshiva UniversityBronx, NY, USA; ^2^Gruss Magnetic Resonance Research Center, Albert Einstein College of Medicine, Yeshiva UniversityBronx, NY, USA; ^3^Department of Radiology, Albert Einstein College of Medicine, Yeshiva UniversityBronx, NY, USA; ^4^Department of Neuroscience, Albert Einstein College of Medicine, Yeshiva UniversityBronx, NY, USA; ^5^Department of Psychiatry and Behavioral Sciences, Albert Einstein College of Medicine, Yeshiva UniversityBronx, NY, USA

**Keywords:** ADHD, brain networks, structural MRI, fMRI, DTI, EEG/ERP

## Abstract

Attention-deficit/hyperactivity disorder (ADHD) is the most commonly diagnosed neurodevelopmental disorder in childhood, which affects more than 5% of the population worldwide. ADHD is characterized by developmentally inappropriate behaviors of inattention, and/or impulsivity and hyperactivity. These behavioral manifestations contribute to diminished academic, occupational and social functioning, and have neurobiological bases. Neuronal deficits, especially in the attention and executive function processing networks, have been implicated in both children and adults with ADHD by using sophisticated structural and functional neuroimaging approaches. These structural and functional abnormalities in the brain networks have been associated with the impaired cognitive, affective, and motor behaviors seen in the disorder. The goal of this review is to summarize and integrate emerging themes from the existing neuroimaging connectivity studies based on advanced imaging techniques, applied in data of structural magnetic resonance imaging (MRI), functional MRI (fMRI), diffusion tensor imaging, electroencephalography and event related potential; and to discuss the results of these studies when considering future directions for understanding pathophysiological mechanisms and developmental trajectories of the behavioral manifestations in ADHD. We conclude this review by suggesting that future research should put more effort on understanding the roles of the subcortical structures and their structural/functional pathways in ADHD.

## INTRODUCTION

Attention deficit/hyperactivity disorder (ADHD) is the most commonly diagnosed neurodevelopmental disorder in childhood. Diagnoses of ADHD are made based on developmentally inappropriate behavioral symptoms, which have been categorized into three subtypes: inattentive, impulsive and hyperactive, and combined type. These core behavioral symptoms must be pervasive across situations, persistent for more than 6 months and observed before the age of 7 years, defined by the diagnostic and statistical manual of mental disorders (DSM-IV-TR) issued by the American Psychiatric Association (2000). The DSM-IV-TR reported that 3–7% of school-aged children have ADHD. However, most recent surveys have estimated significantly increased prevalence rates, from 6.9% in 1998 to 9.0% in 2009, shown in children aged 5–17 years (Akinbami et al., 2011).

Attention deficit/hyperactivity disorder is considered one of the most hereditable disorders with an estimated mean heritability of 75% (Faraone, 2005). Besides the genetic component, ADHD also has neurobiological and environmental underpinnings. The etiology of this highly inhomogeneous disorder is still unknown. Apart from the behavioral symptoms that are used for diagnostic measurements, both children and adults with ADHD have been found to have impairments in neural networks associated with sensory and cognitive processing functions. For instance, neuronal deficits in attention and executive function processing networks have been frequently reported in both children and adults with ADHD, by using sophisticated structural and functional neuroimaging approaches (Bush, 2005; Konrad and Eickhoff, 2010). And these structural and functional abnormalities in the brain have been associated with impaired cognitive, affective, and motor behaviors seen in ADHD approaches (Bush, 2005; Konrad and Eickhoff, 2010). In addition, spontaneous low-frequency functional activities have been reported in multiple brain regions, which formed the default mode network (DMN), during wakeful resting-state functional magnetic resonance imaging (fMRI) acquisition (Bartels and Zeki, 2005). Patients with ADHD have been reported to have both structural and functional abnormalities associated with the DMN (Konrad and Eickhoff, 2010; Castellanos, 2012).

In this study, we will review and summarize these existing studies, which have assessed the regional features and systems-level patterns of the structural and functional brain networks in ADHD, based on advanced computational techniques applied to data of structural MRI, diffusion tensor imaging (DTI), fMRI, electroencephalography (EEG), and event related potential (ERP). We will also discuss the results of these studies when considering future directions for understanding pathophysiological mechanisms and developmental trajectories of the behavioral manifestations in ADHD.

## STRUCTURAL MRI-BASED BRAIN NETWORKS IN ADHD

Structural MRI is the primary imaging tool used for studying brain anatomy and identifying changes in brain structures. Commonly used structural MRI measures include volume and density of the gray (GM) or white matter (WM) of the whole brain or sub-regions, as well as the regional and whole brain cortical GM thickness (Vaidya, 2012). Techniques for investigating the topological and structural features of the anatomical brain networks have also been developed by using regional volume or thickness as basic metric (Lerch et al., 2006; He and Evans, 2007; Zielinski et al., 2010).

The early estimations showed approximately 4–5% overall cerebral and cerebellar volumetric reductions in children and adolescents with ADHD, compared to that of typically developing controls (TDC; Castellanos, 2002; Carmona et al., 2005). Other structural MRI studies have reported volumetric reductions in the frontal lobe, [including orbitofrontal (OFC), superior frontal (SFC), and dorsolateral prefrontal (DLPFC) cortices], posterior and anterior cingulate gyri, precentral gyrus, caudate nuclei, corpus callosum (CC), as well as the cerebellum (Seidman et al., 2005; Shaw et al., 2006; Bush, 2011). Significantly reduced whole brain cortical GM thickness has also been found in children with ADHD when compared to TDC (Shaw et al., 2006, 2007; Makris et al., 2007). Studies also showed significantly thinner cortical thickness in regions including bilateral DLPFC and OFC, anterior and posterior cingulate cortex (PCC) and the temporo-occipito-parietal junction, in adults with ADHD when compared to controls (Makris et al., 2007; Proal et al., 2011). The rate of cortical thinning in these regions has been shown to be inversely associated with the severity of hyperactivity and impulsiveness in normal development (Shaw, 2011).

Basal ganglia regions, such as the globus pallidus, putamen, and caudate have been reported to have structural abnormalities in children with ADHD. Structural MRI studies have detected reduced globus pallidus, putamen, and caudate volumes in voxel-based studies (Frodl and Skokauskas, 2012), and in manual tracing region of interest (ROI)-based deformation analysis (Qiu et al., 2009) in children with ADHD. Interestingly, they did not find any regional volumetric differences of the basal ganglia in adults with ADHD when compared to age-matched controls (Qiu et al., 2009; Frodl and Skokauskas, 2012). However, clinical studies found that the hyperactive/impulsive symptoms, observed in children with ADHD, significantly decline over time, whereas the inattentive symptoms rarely vanish (Lahey et al., 2005). Thus, the similar striatal volumes shown in the adults with ADHD and age-matched control may explain the vanished hyperactivity/impulsivity symptoms during the adulthood in many of the clinical cases. More cross-sectional and longitudinal investigations need to be done, to clarify the relationships among the striatum, its associated brain pathways, and the developmental trajectories of the disorder.

By now, most replicated findings from the voxel-based and ROI-based structural MRI studies have suggested significant decrease of the whole brain GM and WM volumes and significant regional underdevelopment in the prefrontal cortex (PFC), including the OFC, DLPFC, and SFC, basal ganglia substructures (striatum and globus pallidus), and cerebellum, in patients with ADHD (Frodl and Skokauskas, 2012). Structural changes, in the brain networks encompassing PFC and its connections to the striatum and cerebellum, have been found to be associated cognitive impairments, such as distractibility, forgetfulness, impulsivity, poor planning, and locomotor hyperactivity, in both children and adults with ADHD (Seidman et al., 2005; Arnsten, 2006).

From literature, structural MRI-based techniques for constructing the anatomical brain networks, such as in (Lerch et al., 2006; He and Evans, 2007; Zielinski et al., 2010), have not yet been implemented in cohorts with ADHD. Investigations of the topological features and pair-wise nodal communication patterns of the anatomical networks, and their relationships with the clinical and behavioral manifestations are important future directions in the research field related to ADHD.

## FUNCTIONAL BRAIN NETWORKS IN ADHD

Functional MRI techniques provide a way to understand normal brain functions and to test for regional brain dysfunctions associated with disorders (Bush, 2005). Both task-based and resting-state fMRI have been frequently applied in children with ADHD, and have demonstrated atypical functional activations in the frontal, temporal, parietal lobes, and cerebellar regions (Shaw et al., 2006; Cubillo et al., 2010, 2011; Rubia et al., 2010). The frontal cortex can be divided into five major functional sub-regions: the orbital, dorsolateral, mesial (all which make up the PFC), the premotor, and motor regions. Social inhibition and impulse control are associated with the OFC (Fischer et al., 1990). Abnormal functional activations in OFC have been suggested to influence behavioral inhibition in children with ADHD (Bush, 2010). The DLPFC, another most frequently reported region of functional impairment in ADHD, has been implicated in planning, working memory and attentional processes (Danielson et al., 2011). In addition, one fMRI study conducted in adults with childhood ADHD showed reduced activations in bilateral inferior prefrontal cortices (IFC), left parietal lobe, caudate and thalamus, and reduced inter-regional functional connectivity between right inferior fronto-frontal, fronto-striatal, and fronto-parietal neural networks during a stop and switching task, when compared to controls (Cubillo et al., 2010).

Structures of the cingulo-fronto-pariental (CFP) cognitive/attention network, including the fronto-striatal and fronto-parietal pathways, are thought to be the primary substrate for most attention and executive functions (Bush, 2011). The main regions that comprise the CFP network are the lateral frontal pole, dorsal anterior cingulate cortex (dACC), DLPFC, ventrolateral PFC (VLPFC), caudate, cerebellum, and the parietal cortex (Bush, 2010). This network controls goal-directed processes and provides the ability to respond to changing task demands (Castellanos, 2012). Significantly decreased activations have been reported in DLPFC, VLPFC, IFC, and superior parietal cortex (SPC) in ADHD, during multiple cognitive performance tasks and in resting-state (Rubia et al., 2010; Bush, 2011; Castellanos, 2012).

The dACC is an important component of the fronto-striatal circuitry of the CFP network, which has been consistently reported to have abnormal activation in ADHD (Sun et al., 2012). The dACC has a critical role in attention, cognitive processing, target detection, novelty detection, response selection, response inhibition, error detection, and motivation (Bush, 2010). Hypotheses about its functions include reward-based decision-making, response selection, error detection, and predicting task difficulty, which have shown to be impaired in children and adults with ADHD (Seidman et al., 2005). An attention task-based fMRI study found hypo-activation of the dACC in adults with ADHD when compared to controls (Bush, 2011). Resting-state fMRI studies frequently reported disrupted functional connectivity between the dACC and PCC (Castellanos et al., 2008), and abnormal developmental pattern of the dACC–DMN interactions in ADHD subjects (Fair, 2010; Sun et al., 2012). The atypical connectivity in ADHD may relate to delayed or disrupted maturation. ADHD adults presented with abnormal dACC-PCC connectivity patterns when compared to age-matched TD adults. Connectivity patterns were similar between the ADHD group and the young TD subjects, indicating atypical brain maturation in the ADHD group (Sato, 2012). In addition, significantly increased functional connectivity between the dACC and the bilateral thalamus, bilateral cerebellum, and bilateral insula have been shown during resting-state in children with ADHD, compared to TDC (Tian, 2006).

The thalamus is a key subcortical structure of the cortico-striato-thalamo-cortical (CSTC) loops that serve attentional and cognitive processing. Significantly reduced regional activations in bilateral thalami (especially in the pulvinar nuclei), significantly decreased functional connectivity between bilateral pulvinar and right prefrontal regions, and significantly increased connectivity between the right pulvinar and bilateral occipital regions have been reported in children with ADHD, during a visual sustained attention task-based fMRI study (Li et al., 2012). Another study has found reduced functional connectivity between thalamus and basal ganglia areas (especially in putamen) in children with ADHD, during resting-state (Cao et al., 2009).

Altered topological features and inter-regional functional connectivity in large-scale brain networks encompassing cortical and subcortical regions have been increasingly reported, indicating systematic and more widespread brain alterations in ADHD. Resting-state fMRI has been used across laboratories to identify neural networks such as the DMN, dorsal, and ventral attentional networks, as well as motor, visual, and executive control systems (Fox et al., 2006; Buckner et al., 2008; Castellanos, 2012). The DMN is a distributed network of brain regions, which is more active during rest than during performance of sensory and cognitive demanding tasks. Studies have found significantly decreased functional connectivity among the brain regions of the DMN, and between those with putamen and thalamus (Cao et al., 2009; Qiu et al., 2011). Incremental deactivations of the regions in the DMN have been associated with increased task difficulty as well as during transition from rest-to-task states in ADHD (Konrad and Eickhoff, 2010; Liddle, 2011). Furthermore, by applying the graph theoretical approach (GTT), which has been used to characterize the topology of global and regional brain communications (Konrad and Eickhoff, 2010; Ahmadlou et al., 2012), a resting-state fMRI study found significantly increased local efficiency combined with a decreasing tendency in global efficiency of the DMN, and significantly decreased nodal efficiency in the medial prefrontal, temporal, occipital, and subcortical regions in children with ADHD, when compared to the control group (Wang, 2009). Castellanos (2012) have suggested that ADHD could be considered as a DMN disorder. In addition, a resting-state fMRI study, by running network based statistics (NBS) in 90 cortical and subcortical regions, demonstrated abnormal inter-regional connectivity of the frontal-amygdala-occipital network and frontal-temporal-occipital network in young adults with ADHD (Cocchi, 2012). Impaired inter-regional connectivity within reward-motivation regions and their decreased connectivity with regions from the DMN and dorsal attentional networks have also been reported, and suggest impaired interactions between control and reward pathways that might underlie attention and motivation deficits in ADHD (Tomasi and Volkow, 2012).

Altered topological features and inter-regional functional connectivity in large-scale brain networks in ADHD, which have been reviewed in the fMRI section, are convinced in EEG/ERP studies as well. A GTT-based study in resting-state EEG data reported abnormal cluster coefficients and path lengths of the nodes in the left hemisphere, which were recognizable in the delta band, in patients with ADHD when compared to controls (Ahmadlou et al., 2012). An NBS study in sustained attention task-based EEG data showed distinct frontal-central-parietal patterns in the theta and alpha frequency bands in adults with ADHD compared to controls, where ADHD subjects displayed a more robust homogeneous response pattern in the 120–260 ms time range that included the P1, N1, P2 component, with a majority of latency peaks characterized by alpha and theta activation in the fronto-central sites (Shahaf et al., 2012). Similar findings have been interpreted as revealing a compensatory mechanism activated by ADHD patients in early stages of stimulus processing, by which more attention was directed to the task (Prox et al., 2007).

## DTI-BASED BRAIN NETWORKS IN ADHD

While functional brain imaging studies may reveal specific regions of dysfunction within the brain, it is important to know how the nodes within these networks are structurally connected. Micro-structural abnormalities in the WM may lead to disrupted functional communications between brain regions, ultimately resulting in disrupted behavioral functioning in ADHD (Nagel, 2011). DTI is an MRI method that provides *in vivo* information about the WM microstructure through water diffusion, which can reveal microscopic details about tissue architecture (Konrad and Eickhoff, 2010). Orientations of the WM tracts in the brain can be measured by the directions of diffusivity (Konrad and Eickhoff, 2010). The most common quantitative indices used to measure the WM integrity are mean diffusivity (MD) and fractional anisotropy (FA; Konrad and Eickhoff, 2010; Nagel, 2011).

Two primary analysis methods have been applied in DTI studies: voxel-based analysis (VBA) that allows for a complete overview of the WM integrity in the brain, and ROI-based analysis for more specific exploration of the WM abnormalities in certain brain regions. A recent meta-analysis reviewed the ROI-based studies assessing the WM integrity, and provided evidence of several disturbed WM regions in children with ADHD, including the inferior and superior longitudinal fasciculus, anterior corona radiate, cortico-spinal tract, cingulum, CC, internal capsule, caudate nucleus, and cerebellum (van Ewijk, 2012). Review of the VBA studies also confirmed WM changes in these regions, and found extensive differences across the four brain lobes, as well as areas within the basal ganglia, uncinate fasciculus, and forceps minor (van Ewijk, 2012).

Development of WM determinative and probabilistic tractography techniques has made it possible to estimate and visualize the structural connectivity of the WM pathways in human brain. Using tractography-based analyses, DTI studies have demonstrated increased FA in WM structures connecting parietal-occipital regions (Silk, 2009), and tracts connecting the temporal lobe and other distant cortical regions in children with ADHD compared to TDC, which were positively associated with symptom severity in the patient group (Peterson, 2011). Significantly reduced FA in the cortico-spinal tract (Carmona et al., 2005; Hamilton et al., 2008; Cubillo, 2010), the superior longitudinal fascicle that connects the prefrontal and parietal regions (Makris, 2008; Cubillo et al., 2010; Konrad, 2010), the cingulum bundle (Makris, 2008; Konrad, 2010), have also been reported in patients with ADHD. In addition, one study detected significantly increased MD in the frontal portion of the left fronto-occipital fasciculus in adults with ADHD when compared with controls (Konrad et al., 2006). DTI studies have also shown alterations within the cerebellar WM areas in children and adolescents with ADHD (Ashtari et al., 2005; Bechtel et al., 2009).

The neural pathways that are associated with the areas of abnormal WM reviewed above are the pathways connecting the cortical regions, cortical-striatum and cortical-cerebellum. The prevailing theory regarding the neurobiological basis of ADHD identified the fronto-striatal network as a probable substrate of cognitive and behavioral impairments seen in ADHD (Bush, 2005; van Ewijk, 2012). Studies found disturbed WM structural connectivity of the frontal-striatal network in both adults and children with ADHD, compared to group-matched controls (Konrad and Eickhoff, 2010; Tamm et al., 2012). Tract- specific analyses found reduced FA in bilateral fronto-striatal fiber tracts in children with ADHD, specifically in the orbitofrontal and ventrolateral tracts, associated with poor executive functioning performance (Shang, 2013). In addition, significant reductions of probabilistic WM connectivity between the thalamus and striatum has been reported in children with ADHD when compared to TDC (Xia et al., 2012).

The CC, the largest band of WM fibers in the brain that connects the left and right hemispheres, plays an important role in inter-hemispheric communication. Thus, abnormal microstructure of CC may affect cognitive functions that depend on bilateral collaboration (Vaidya, 2012). Across studies, children with ADHD showed reduced volume of the splenium, the posterior region of the CC that connects bilateral parieto–temporal cortices (Valera, 2007). Using both DTI and anatomical MRI, one study found microstructure abnormalities in the isthmus/splenium part of the CC, characterized by reduced FA values in adults with ADHD when compared to healthy controls (Dramsdahl, 2012). These results are in line with two earlier studies, which observed reduced FA in the isthmus in children with ADHD (Chao, 2009; Cao, 2010).

## DISCUSSIONS

Attention-deficit/hyperactivity disorder is the most common neurodevelopmental disorder in childhood. Neuroimaging studies have attempted to identify the pathophysiology of the disorder by searching for abnormalities in brain regions and their connections that are involved in attention, executive function, motor control, response inhibition, working memory, and even during rest. We reviewed and summarized the important findings from the structural MRI, fMRI, EEG/ERP, and DTI studies, which have provided the abundant evidence of structural and functional alterations in widespread brain regions and their connections, in this severe and heritable brain disorder.

The majority of the existing neuroimaging studies, which have attempted to find the neurobiological underpinnings of ADHD, have focused on cortical regions and their connections, and has demonstrated global cortical maturation delay based on reduced cortical thickness and reduced GM and WM volumes, specifically in frontal lobe (Carmona et al., 2005), regional WM micro-structural abnormalities in frontal, temporal and parietal lobes (Nagel, 2011; Dramsdahl, 2012; Shang, 2013), and aberrant neuronal activations, inter-regional functional connectivity and global network features over these cortical areas, during sensory and cognitive performance or even at rest (Wang, 2009; Ahmadlou et al., 2012; Castellanos, 2012; Cocchi, 2012; Shahaf et al., 2012). Furthermore, the existing studies also suggest that the structural and functional connectivity deficits and the ADHD symptoms might arise incidentally from a common etiologic mechanism, involving altered modulation of synaptic potentiating and pruning by dopamine and other factors during development, which result in altered patterns of cortico-cortical connectivity that might persist into adulthood (Liston et al., 2011).

Subcortical regions may also significantly contribute to the pathophysiology of ADHD. For example, the basal ganglia has been associated with the execution of appropriate goal-directed behaviors and may play a role in the behavioral impairments for response control seen in many children with ADHD (Qiu et al., 2009). Neuroimaging studies have demonstrated regional structural and functional deficits of the basal ganglia, especially in the striatum (Qiu et al., 2009; van Ewijk, 2012). Disturbed WM structural connectivity and atypical functional connectivity have been shown in the frontal-striatal network in both adults and children with ADHD (Konrad and Eickhoff, 2010; Tamm et al., 2012). It has been hypothesized that the impairments of the striatum and its brain connections are associated with the hyperactivity/impulsivity component in children with ADHD (van Ewijk, 2012).

However, the role of the thalamus and its mediating role in cortico-striatal and cortico-cortical pathways in ADHD have been relatively ignored. Very recently, investigation of high resolution structural MRI data revealed reduced bilateral thalamic volumes, as well as regional surface atrophy in the pulvinar nucleus of the left side thalamus in children with ADHD (Xia et al., 2012). In the same study, disturbed frontal-thalamo and thalamo-striatal WM connectivity have also been demonstrated in the children with ADHD. Furthermore, significantly reduced pulvinar activations and abnormal pulvinar-frontal and occipital-pulvinar functional connectivity have been shown in children with ADHD during a visual sustained attention task, which were also significantly correlated with their inattentiveness indices for clinical diagnoses (Li et al., 2012). This series of neuroimaging studies may drive the field forward by placing the pulvinar nuclei of the thalamus at the center of dysfunctional attentional networks in ADHD (Shaw, 2012).

As a summary, brain network associated dysfunctions have been found to be central in ADHD pathophysiology. It is important to gain a better understanding of how subcortical-cortical and cortical-cortical networks development is altered during the onset of the disorder. Thus, future studies should allocate greater resources on subcortical regions and relationships between subcortical and cortical regions in order to provide a better understanding of the etiology of the disorder.

## Conflict of Interest Statement

The authors declare that the research was conducted in the absence of any commercial or financial relationships that could be construed as a potential conflict of interest.
